# An Optical Section-Assisted *In Vivo* Rabbit Model for Capsular Bend and Posterior Capsule Opacification Investigation

**DOI:** 10.1371/journal.pone.0148553

**Published:** 2016-02-03

**Authors:** Pingjun Chang, Lei Lin, Qian Zheng, Fang Yu, Xiaoyu Yu, Yinying Zhao, Xixia Ding, Weigen Zhu, Jin Li, Yun-e Zhao

**Affiliations:** School of Ophthalmology and Optometry, Wenzhou Medical University, Wenzhou, Zhejiang, China; Bascom Palmer Eye Institute, University of Miami School of Medicine;, UNITED STATES

## Abstract

**Purpose:**

To establish an optical section-assisted in vivo rabbit model for capsular bend and posterior capsule opacification (PCO) investigation.

**Methods:**

A total of 10 rabbits underwent phacoemulsification surgery and intraocular lens (IOL) implantation. On the basis of the relationship between the anterior capsule and IOL, the rabbits were divided into complete overlap and incomplete overlap groups, in which six and four rabbits were included, respectively. The capsular bend optical sections were assessed using ultra-long scan depth optical coherence tomography (UL-OCT), and posterior capsule opacification was evaluated with slit lamp on postoperative day 3, 7, 14, and 28. In addition, histopathological section was used to verify the accuracy of capsular bend type captured by OCT in three rabbits.

**Results:**

Based on the special animal model, six capsular bend types were observed, namely, anterior (A), middle (M), posterior (P), detachment (D), funnel (Fun) and furcate adhesion (Fur). On day 3, capsular bend began to form. On 14 days, the capsular bends were comprised of A, M and D types, which were almost maintained until day 28. Histopathological section findings were consistent with optical sectioning results. In the incomplete and complete groups, the earliest PCO within the optical zone were on day 7 and 28, respectively. The incomplete group exhibited higher incidence and faster PCO on day 7 (p = 0.038) and 14 (p = 0.002).

**Conclusions:**

This animal model not only mimics capsular bend evolution and PCO processes but also produces OCT optical section images equivalent to and more repeatable than histopathology, thereby providing a promising method for the further investigations of PCO.

## Introduction

Posterior capsule opacification (PCO) results from the migration and proliferation of residual lens epithelial cells (LECs) is the main cause of secondary visual loss following cataract surgery. The incidence of PCO in children is nearly 100%. [1] The capsular bend represents the space configuration among the anterior capsule, posterior capsule, and intraocular lens (IOL). Previous studies [2–6] demonstrated that the capsular bend induced by IOL with a sharp optic edge can prevent PCO.

However, the underlying mechanism of how sharp edge-designed IOL prevents PCO remains controversial. Nishi et al [3] reported that capsular bends which acted like an mechanical barrier, inhibit LECs proliferation and migration through contact inhibition effect. Nevertheless, Nagamoto et al [7] emphasized that the compression between IOL and capsular bag plays an important role in PCO prevention.

Nishi et al [3–6] established an animal capsular bend model and concluded by histopathological sections that the LECs created by a sharp optical edge at the capsular bend are significantly inhibited. However, certain limitations are inevitably existed when this method is applied. First, an active observation of the capsular bend evolution is impossible. Second, previous clinical study findings [8] indicated various capsular bend types; however, whether these types playing a crucial role in PCO prevention remains unknown. Previous animal models obviously cannot satisfy the demands of capsular bend study. Thus, the establishment of a new animal model for further research on capsular bends and PCO mechanisms is a worthwhile endeavor.

On the basis of the transparent characteristics and different optical properties of the cornea, capsule and IOL, ultra-long scan depth optical coherence tomography (UL-OCT) with high axial resolution can capture accurate and clear optical sectioning at the capsular bend. Therefore, an animal model was designed using this optical section method to investigate the configuration of the capsular bend and the PCO incidence after cataract surgery, as well as to evaluate the evolution of the capsular bend–IOL complex. With the purpose to make repeated measurements possible and provide a promising approach for further PCO studies.

## Materials and Methods

### 2.1 Animal model

#### 2.1.1 Ethics statements

A total of 10 three months old Japanese white rabbits (Laboratory Animal Centre of Wenzhou Medical University, Wenzhou, Zhejiang, China) were used in this study. The animal experiments were approved by the Laboratory Animal Ethics Committee of Wenzhou Medical University (File number: wydw2013-0020) and consistent with the ARVO Statement for the Use of Animals in Ophthalmic and Visual Research. The study was performed in strict accordance with standard surgical procedures and all efforts were made to ameliorate animal suffering.

#### 2.1.2 Surgical procedures

The surgical procedures were strictly followed. One experienced surgeon (Jin Li) performed all the surgeries under intramuscular anesthesia with 3% pentobarbital sodium (1 mL/kg, Sile Instrumentation Company, Guangzhou, China) combined with compound narcotic Su-Mian-Xin (0.1 mL/kg, Shengda Pharmaceutical Co., Limited, Jilin, China). The following standard phacoemulsification technique was utilized: first, a 2.2-mm clear corneal incision was performed at 11 o’clock in the right eye, and a continuous curvilinear capsulorhexis was created using capsule forceps. After hydrodissection, phacoemulsification and cortex aspiration, an IOL (Alcon, SN60AT, 30D) was implanted into the capsular bag and the haptics of the IOL were perpendicular to the line of inner and outer canthus. The incisions were closed with 10–0 nylon sutures. Levofloxacin (Santen, Japan) and tobramycin dexamethasone (Alcon, USA) eye drop or ointment were used for anti-infection and anti-inflammatory after surgery.

#### 2.1.3 Intraocular lens

The IOL used in this study was the sharp edge-designed SN60AT (Alcon, USA) with 30D power. Although the rabbit capsule was larger than the human capsule, the IOL with 13.0 mm haptic length and nice adhesion characteristics was used in this study to reduce the maximum extent of effects among different species.

### 2.2 Observation of the capsular bend-IOL complex

#### 2.2.1 UL-OCT

The UL-OCT instrument has been thoroughly described in previous studies. [8–10] In brief, custom-built UL-OCT with 7.5 μm axial resolution and 7.8mm scan depth was used in this study to evaluate the capsular bend. Examinations were postoperatively performed on day 3, 7, 14 and 28. Up to 1% tropicamide and 2.5% phenylephrine hydrochloride were used to dilate the pupil to at least 6.5 mm. The scan width was then set to 12 mm, and the X axis and Y axis were simultaneously aligned to the apex. After adjusting the scan position perpendicular to the haptic line, the nasal and temporal sides of the capsular bend-IOL complex images were captured. The superior side was identified as temporal side when the haptic line was horizontal.

#### 2.2.2 Slit lamp

After the UL-OCT examination, the anterior segment was photographed via slit lamp, and its condition was examined in terms of anterior chamber inflammation and anterior and posterior capsules. Posterior capsule opacification was assessed using retroillumination photographs. Once observed posterior capsule opacity within the IOL optic edge, PCO positive were recorded.

#### 2.2.3 Histopathological section

On day 11 postoperatively, three rabbits were sacrificed by overdose injection of 3% pentobarbital sodium (2 mL/kg). After enucleation, the capsular bend-IOL complexes were sufficiently fixed and dehydrated in 70%, 85%, 95%, and 100% ethyl alcohol for 1, 1, 2, and 3 h, respectively. After being washed with distilled water, the specimens were embedded with Technovit 7100 (T7100) (Heraeus Kulzer, Germany) in accordance with the manufacturer’s instructions, as follows. First, the specimens were placed in liquid A, which contained 100 mL of T7100 and 1 g of hardening agent I, for 1 h. The specimens were then washed twice with distilled water and placed in liquid B, which contained 15 mL of liquid A and 1 mL of hardening agent II for 12 h. The specimens in the scan position were sliced into 10 to 13 μm-thick sections and observed under a microscope after being stained with toluidine blue.

### 2.3 Statistical analyses

Data were analyzed using SPSS 18.0 software. Fisher’s exact test was used to compare the differences between two groups. P values below 0.05 were considered statistically significant.

## Results

### 3.1 Rabbit

**Fig 1:**
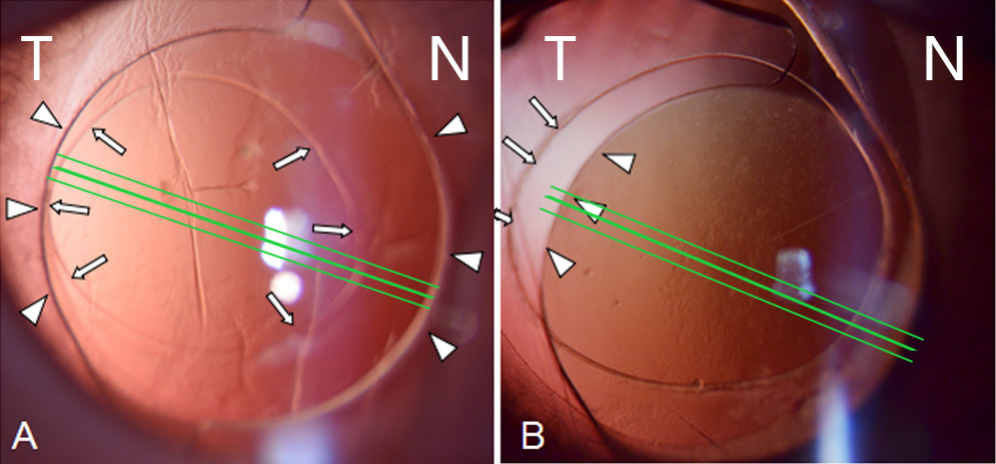
Schematic of relationship between anterior capsule (white arrow) and IOL optic edge (white triangle) in the scan position (green lines). N stand for nasal side, while T stand for temporal side. (A) The temporal side illustrates an undesirable overlap (the capsule overlap less on the IOL optic edge), and the right side illustrates a complete overlap (the capsule complete overlap the IOL in the scan position). (B) The temporal side capsule does not overlap the IOL optic.

**Fig 2:**
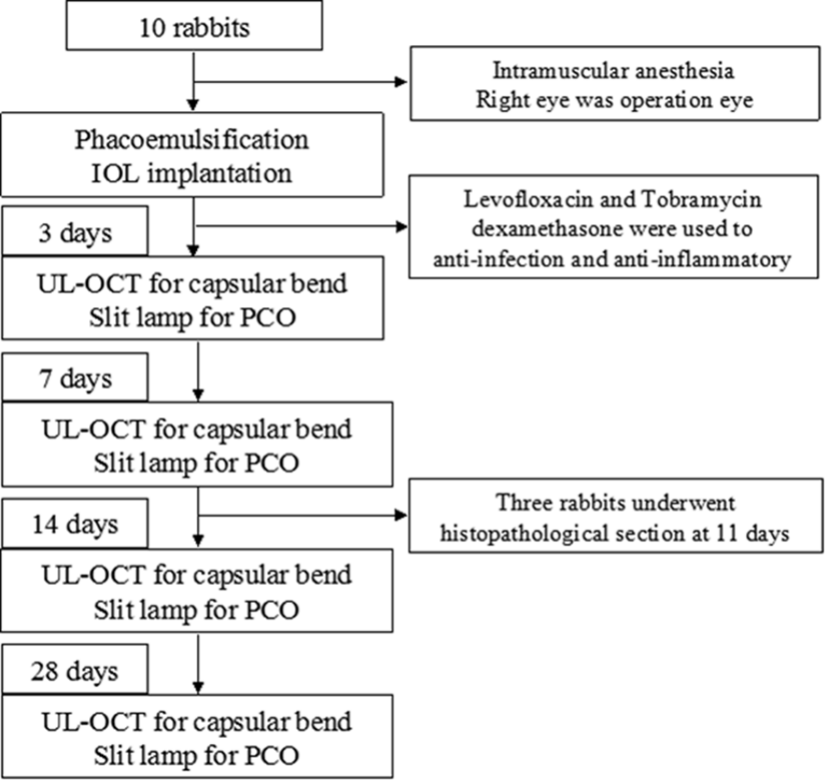
Flow chart of the experiment. Levofloxacin and tobramycin dexamethasone eye drop or oculentum were used for anti-infection and anti-inflammatory for at least 7 days after surgery.

Based on the relationship between the anterior capsule and IOL, the rabbits were divided into complete overlap group (CO group, i.e., the anterior capsular edge completely overlapped with the IOL optic edge) and incomplete overlap group (ICO group, i.e., the anterior capsule did not cover the IOL optic edge or showed significantly eccentric overlap), which comprised six and four rabbits, respectively. Detailed images are shown in Fig 1, and a flow chart that summarizes the experiment is illustrated in Fig 2. Three out of the 10 rabbits were sacrificed on day 11 for histopathological sectioning in the OCT scan position. In addition, the pupils of the rabbits cannot be sufficiently dilated in some instances. Thus, the numbers of rabbit eyes captured were inconsistent across each examination time point.

### 3.2 Capsular bend type

**Fig 3:**
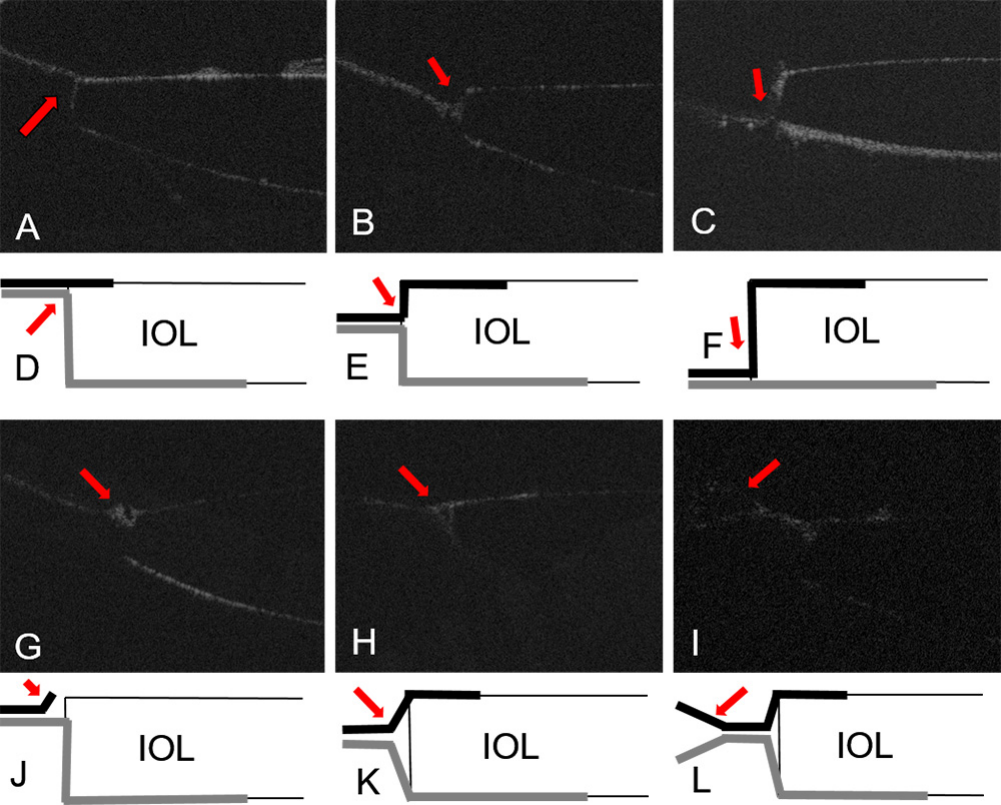
UL-OCT images (A-C & G-I) and corresponding schematic (D-F & J-L) of capsular bend types. Red arrows demonstrate the characteristics of different capsular bend types. In schematic images, black and gray lines represent anterior and posterior capsules, respectively. (A and D) Anterior adhesion type: the posterior and anterior capsule form a stable adhesion at the anterior surface of the IOL optic edge. (B and E) Middle adhesion type: the posterior and anterior capsule wrapped around the IOL and met at the middle of the IOL edge. (C and F) Posterior adhesion type: the anterior and posterior capsule form a stable adhesion at the posterior surface of the IOL. (G and J) Detachment type: the anterior capsule detached from the IOL optic edge. (H and K) Funnel adhesion type: the posterior and anterior capsules adhered to each other, but a space between the capsule and IOL remained and showed the appearance of a funnel. (I and L) Furcate adhesion capsule type: the inner sides of the posterior and anterior capsules adhered, whereas the peripheral side was separated.

During the postoperative observation, the following six types of capsular bend were identified: anterior adhesion (A), middle adhesion (M), posterior adhesion (P), detachment (D), funnel adhesion (Fun) and furcate adhesion (Fur). The UL-OCT images and corresponding schematic of these types are presented in Fig 3.

### 3.3 Histopathological section

**Fig 4:**
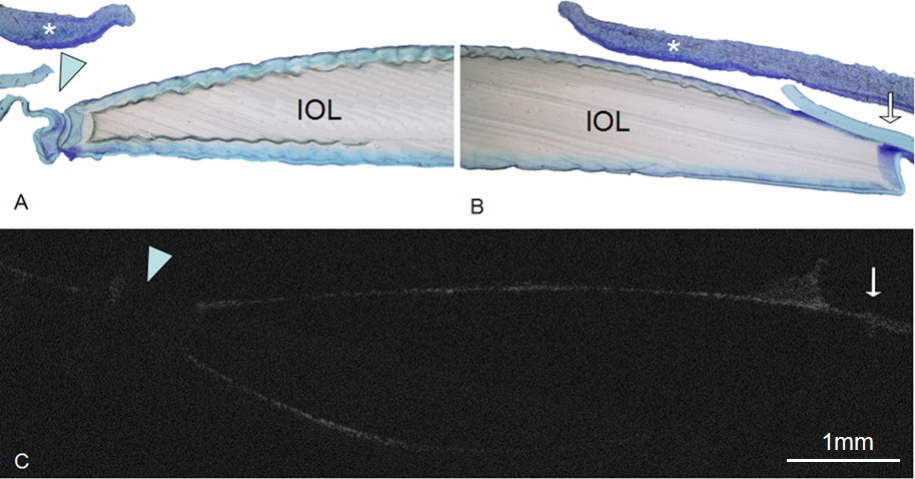
Histopathological section observations on day 11 postoperatively and OCT images on day 7 postoperatively at the scan position. (A) Unformed capsular bend configuration at temporal side (blue triangle). (B) Anterior adhesion type at nasal side (white arrow). (C) OCT image of the same rabbit showing the same capsular bend configuration at the same place. The white asterisk indicates the iris.

The histopathological section findings acquired after 11 days revealed anterior capsular bend type and unformed capsular bend configurations, which were consistent with optical sectioning results. Sample results from one of these rabbits are displayed in Fig 4.

### 3.4 Capsular bend evolution

**Fig 5:**
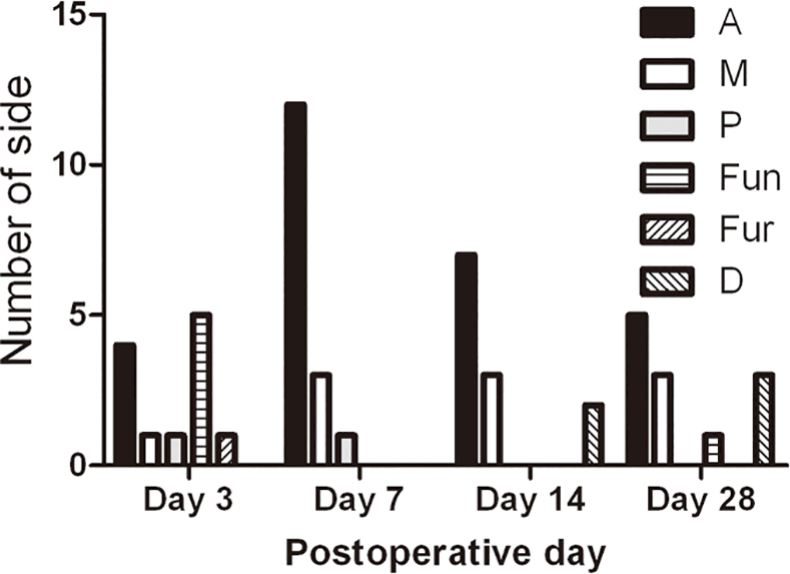
Different types of capsular bends at various times after surgery. A = anterior adhesion type, M = middle adhesion type, P = posterior adhesion type, Fun = funnel adhesion type, Fur = furcate adhesion type, and D = detachment type.

**Fig 6:**
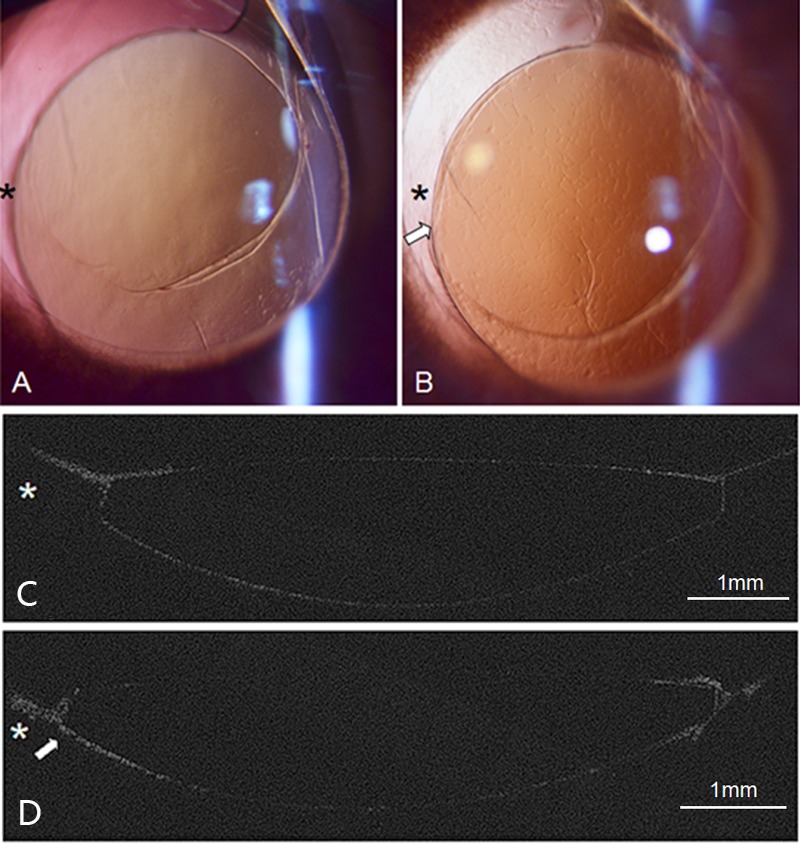
Slit lamp image of a posterior capsule and an OCT image of capsular bend of an incompletely overlapped eye 7 days (A and C) and 28 days postoperatively (B and D). (A and C) On day 7 postoperatively, the capsule was smooth, no opacity was present, and both sides formed anterior adhesion types. (B and D) On day 28 postoperatively, the temporal side (black and white asterisk) exhibited an undesirable overlap, abundant proliferation, and migration of LECs into the posterior capsule. The detachment and funnel adhesion types emerged at the temporal and nasal sides, respectively. Increased thickness of the posterior capsule (white arrow) was apparent in both slit lamp and OCT images.

Each eye with two sides was observed in this study. The number of each capsular bend type at each observation time is demonstrated in Fig 5. On the postoperative day 7, the Fun type transformed into A or M type. Interestingly, on day 14 after surgery, the D types that evolved from A and P types were found on two undesirable overlap sides. The capsular bends of the complete overlap sides were nearly maintained on day 28 postoperatively. However, one A type transformed into a Fun type on an undesirable side (Fig 6). The capsular bends were classified into incomplete adhesion (Fun and Fur), complete adhesion (A, M, and P), and special adhesion (D) types based on the configuration of the characteristic of their evolution. The capsular bend evolutions of the six rabbits that survived for 28 days with sufficient pupil dilation are listed in Table 1.

**Table 1:** Different capsular bend types of six rabbits at two sides (nasal and temporal) postoperatively.

Rabbit	Side	Day 3	Day 7	Day 14	Day 28
R1	Nasal	Un	A	A	A
R1	Temporal	Un	A	D	D
R2	Nasal	A	A	A	A
R2	Temporal	NULL	A	A	A
R3	Nasal	A	M	M	M
R3	Temporal	M	M	M	M
R4	Nasal	Un	A	A	A
R4	Temporal	Fun	M	M	M
R5	Nasal	A	A	A	Fun
R6	Temporal	A	A	A	D
R6	Nasal	Fun	A	A	A
R6	Temporal	P	P	D	D

NULL means UL-OCT observations were hindered by unfavorable mydriasis. Un = unformed capsular bend, A = anterior adhesion type, M = middle adhesion type, P = posterior adhesion type, D = detachment type, and Fun = funnel adhesion type.

### 3.5 Relationship between capsular bend stability and PCO

**Fig 7:**
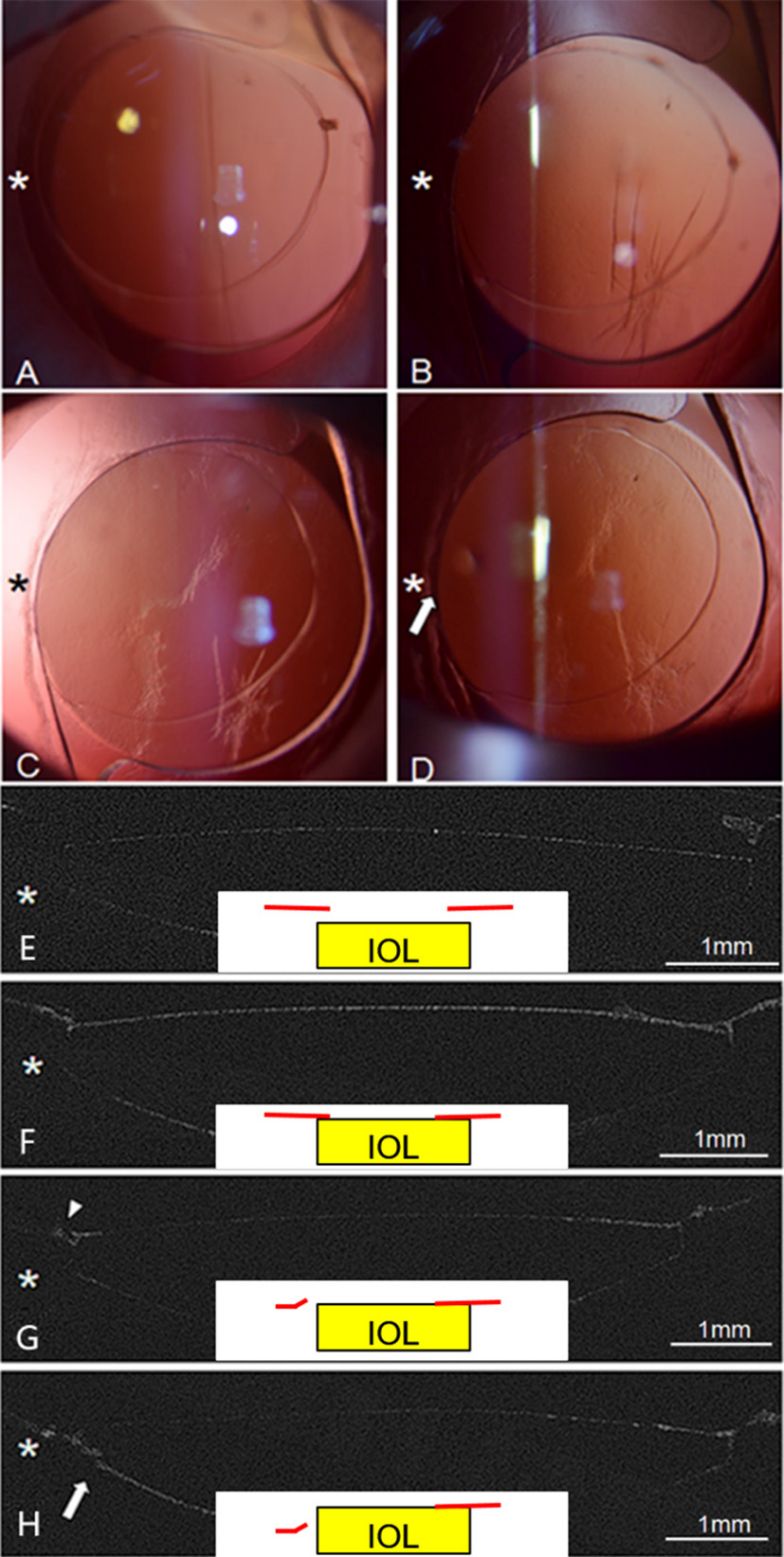
Observation of capsular bend via slit lamp (A-D) and UL-OCT optical sections (E-H) with schematic images embedded in the center of the images. Red lines stand for anterior capsule and yellow squares for IOLs. (A and E) On day 3 postoperatively, an incomplete overlap, anterior capsule not overlapping the IOL at 9 o’clock to 11 o’clock temporally existed, and no opacity were found in the posterior capsule. Neither nasal nor temporal side formed a capsular bend. (B and F) On day 7 postoperatively, the temporal side of the anterior capsule came close to the IOL, and the capsule partially wrapped around the optic edge because of posterior capsular contraction. LECs migrated into the center of the temporal posterior capsule. Both sides formed anterior adhesion types. (C and G) On day 14 postoperatively, the LECs elongated and integrated into a fibrous-like membrane. The anterior capsule detached from the temporal IOL optic edge (white triangle) and transformed into the detachment type, whereas the nasal side remained the anterior adhesion type. (D and H) On day 28, the entire posterior capsule was covered by LECs, and exacerbation of the posterior capsular contraction and increased thickness of the posterior capsule (white arrow) were present. The temporal and nasal sides remained the anterior detachment and anterior adhesion types, respectively.

The earliest posterior capsule opacity within the IOL optic zone were found on day 7 in the incomplete group and 28 in the complete group (Table 2). Five sides of the 12 incomplete sides showed PCO positive on 7 day after surgery, whereas none of the six complete sides presented such opacity, and this difference was significant (p = 0.038). Furthermore, after 14 days postoperatively, all the eight incomplete sides but not the four complete sides exhibited PCO positive; this difference was also significant (p = 0.002). Incomplete overlaps were more prone to form unstable capsular bend that led to the early emergence of PCO. An integrated process of a capsular bend and a PCO of the most typical rabbit are illustrated in Fig 7.

**Table 2:** Number (%) of sides showing PCO in the two groups.

Groups	Day 3	Day 7	Day 14	Day 28
Incomplete overlap group	0	7(58.3%)	8(100%)	8(100%)
Complete overlap group	0	0	0	4(100%)

## Discussion

PCO is a major complication after cataract surgery, which is related to numerous factors, [2, 11–15] including the material, [16–18] biocompatibility [18] and design of the IOL. [2, 5, 19–21] Several studies [3–6, 13] have proven that sharp-edge designed IOL can significantly reduce the incidence of PCO. Previous researches on capsular bend mainly focused on human and animal models in vitro. However, the differences in capsular bend formation between humans and animals remain unknown.

Previous studies on human capsular bends have primarily used slit lamp or Scheimplflug techniques, [13, 22], whereas early OCT research[23, 24] was limited by axial resolution, and the images obtained were not sufficiently clear. Zhao Y et al [8] first used UL-OCT with high axial resolution to investigate the different capsular bend-IOL complex evolutions in highly myopic and emmetropic eyes during the early postoperative period. Nevertheless, clinical studies entail many restrictions precluding the use of in-depth histopathological and cytological experiments. Nishi et al [3–6] established a capsular bend animal model and reported beneficial morphological findings based on histopathological sections that contributed to the understanding of PCO; however, classifications and evolution of capsular bends could not been performed in their study. Therefore, the present animal model in vivo is designed to identify additional details in the observations and provide an efficient approach to investigate the mechanisms of capsular bend formation and LECs behavior.

First, this animal model showed its capability of reliable and repeating observation of capsular bend types. Sacu and coauthors [23] classified four capsular bend configurations by slit lamp, namely, Y, parallel, right-angle and wrapping capsular bend configuration, in which R configurations (same as the anterior capsular bend type in our study) was the most common type. While Zhao Y and coauthors [8] identified six capsular bend types by UL-OCT, namely, anterior adhesion, middle adhesion, posterior adhesion, funnel adhesion, furcate adhesion and parallel adhesion, during their investigation of patients with different axial lengths after cataract surgery. They also found that anterior capsular bend type was the most common type. Our finding performed in animal was similar to Zhao Y et al. [8] Meanwhile, we performed histopathological section and its findings at the scan position were consistent with the UL-OCT. It was interesting that the detachment type that transformed from anterior or posterior capsular bend types was firstly identified in this study, which might be involved with the anterior capsule overlap condition and capsule contraction.

Second, our animal model was able to perfectly mimic the capsular bend evolution as well as the PCO process. Many researchers have described various stage of capsular bend formation. [22–24] Although their descriptions were different, their findings indicated that capsular bend types were in evolutionary states and they were not pertinent during their formation. Zhao Y et al [8] observed that the parallel type can transformed into the Fun, and then transformed into the A type, which was consistent with the capsular bend formation stage theory. Interestingly, in this animal model, we observed that the A and P types might transform into D type, and the A type even occasionally converted into Fun type (Fig 6) which may result from capsule contraction. Furthermore, the speed of capsular bend formation and the development of PCO were closely related. Sacu et al [23] found that one-piece acrylic IOL could develop stable capsular bends after 10 days, but this development was observed on day 7 in the present study, which was much earlier than that observed in humans. [23, 24] Nishi et al [3] investigated the LECs at the capsular bend through immunohistochemical staining with Ki-67 antibody following surgery and revealed that the contact inhibition of the LECs was induced once the capsular bend formed. If the capsular bend formed after LECs migration, it could not exert its contact inhibition effect, and the LECs proliferated and rapidly migrated into the posterior capsule.

In addition, another promising aspect of our model was that it could investigate the relationship of capsule overlap and PCO prevention. Previous study has reported that desirable overlap of the anterior capsule is beneficial to the stable formation of capsular bends and prevention of PCO. [25] In the present study, PCO were observed in the incomplete overlap sides on day 7 after surgery, which was sooner than that in the complete group. Moreover, capsular bend configurations of the completely overlapped eyes did not change on day 7 after surgery, so this finding might be ascribed to the stable formation of the capsular bend. It was postulated that overlapping conditions of the anterior capsule and delayed capsular bend barrier destruction may exerted devastating influences on the stability of the capsular bends. Less extensive investigation has been performed in capsular bend association with PCO. Whether the speed of capsular bend formation or the stability exerted a certain effect on PCO prevention deserve further investigation and is a worthwhile unanswered question.

In summary, the main advantages of the present animal model were derived from the optical section functionality of UL-OCT in animal experiments. First, observations of capsular bend configurations and monitoring can be performed in vivo. Second, the capsular bag could be extracted at any desired time to investigate the cytoskeleton and cell behavior of LCEs at different stages by immunohistochemical or fluorescence staining. Some experiments related to fluorescence staining and preparations of the contact inhibited LECs at the capsular bend have recently been performed and have achieved some progress. Nonetheless, additional studies are needed.

However, several potential limitations of this experiment should be pointed out. First, the slit lamp and UL-OCT observations should be conducted after dilating the pupil to at least 6.5 mm. However, the rabbit pupil cannot always be dilated large enough. Hence, observations may sometimes be hindered by unfavorable mydriasis. Second, only two sides were scanned rather than all dimensions because of time constraints; however, the mutual effects of both sides and the evolution of the entire bag cannot be ignored. Moreover, the spherical lens and large capsular bag of the rabbit resulted in mismatches between the IOL and the capsular bag that led to IOL rotation and shifts in the early period following surgery. Thus, the largest IOL with haptic length of 13.0 mm and perfect adhesion characteristics were used to reduce the maximum degree of effect among different species.

In conclusion, the application of this optical-section assisted capsular bend animal model in vivo not only mimics capsular bend evolution and PCO but also produces OCT optical section images equivalent to and more repeatable than those acquired with histopathology. Thus, this model provides a promising method for further investigations of PCO.

## Supporting Information

S1 Table Different capsular bend types and PCO of the whole rabbits at two sides (nasal and temporal) postoperatively. (DOC)
