# Supplementary material for: An Optical Section-Assisted In Vivo Rabbit Model for Capsular Bend and Posterior Capsule Opacification Investigation
Source: PLoS One. 2016 Feb 3;11(2):e0148553. doi: 10.1371/journal.pone.0148553 (PMC4739694; doi:10.1371/journal.pone.0148553)
Supplement: S1 Table — (DOC) [file pone.0148553.s001.doc]

S1 Table. Different capsular bend types and PCO of the whole rabbits at two sides (nasal and temporal) postoperatively

| Rabbit | Overlap | Day 3 | | Day 7 | | Day 14 | | Day 28 | |
| --- | --- | --- | --- | --- | --- | --- | --- | --- | --- |
|  |  | N | T | N | T | N | T | N | T |
| R1 | ICO | F | Un | A(+) | N(+) | NULL | NULL | NULL | NULL |
| R2 | ICO | Un | NULL | N | A | NULL | NULL | NULL | NULL |
| R3 | CO | F | Un | A | A | NULL | NULL | NULL | NULL |
| R4 | CO | F | Fur | NULL | NULL | NULL | NULL | NULL | NULL |
| R5 | CO | A | M | M | M | M | M | M(+) | M(+) |
| R6 | CO | Un | F | A | M | A | M | A(+) | M(+) |
| R7 | ICO | A | A | A | A | A(+) | A(+) | F(+) | D(+) |
| R8 | ICO | A | NULL | A | A(+) | A(+) | A(+) | A(+) | A(+) |
| R9 | ICO | Un | Un | A(+) | A(+) | A(+) | D(+) | A(+) | D(+) |
| R10 | ICO | F | P | A(+) | P(+) | A(+) | D(+) | A(+) | D(+) |

N = Nasal side, T = Temporal side, CO = complete overlap, ICO = incomplete overlap, NULL means UL-OCT observations were hindered by unfavorable mydriasis. Un = unformed capsular bend, A = anterior adhesion type, M = middle adhesion type, P = posterior adhesion type, Fun = funnel adhesion type, Fur = furcate adhesion type, and D = detachment type. (+) = PCO positive. R1-R3 were sacrificed for histopathological section. The pupil of R4 was unable to dilate after Day 3 and failed to perform the histopathological section.
